# Transfusion burden in early childhood plays an important role in iron overload in Diamond‐Blackfan anaemia

**DOI:** 10.1002/jha2.524

**Published:** 2022-08-30

**Authors:** Jonathan R. A. de Wilde, Birgit van Dooijeweert, Annelies J. van Vuren, Elise J. Huisman, Frans J. Smiers, Arian van der Veer, Richard van Wijk, Wouter W. van Solinge, Edward E. S. Nieuwenhuis, Eduard J. van Beers, Marije Bartels

**Affiliations:** ^1^ Central Diagnostic Laboratory‐Research University Medical Centre Utrecht Utrecht University Utrecht The Netherlands; ^2^ Department of Paediatric Haematology University Medical Centre Utrecht Utrecht University Utrecht The Netherlands; ^3^ Centre for Benign Haematology Thrombosis and Haemostasis Van Creveldkliniek University Medical Centre Utrecht Utrecht University Utrecht The Netherlands; ^4^ Department of Paediatric Haematology Erasmus Medical Centre Rotterdam The Netherlands; ^5^ Department of Paediatric Haematology Leiden University Medical Centre Leiden Leiden The Netherlands; ^6^ Department of Paediatric Haematology Maastricht University Medical Centre Maastricht The Netherlands; ^7^ Department of Paediatric Haematology Amalia Children's Hospital RadboudUMC Nijmegen The Netherlands

**Keywords:** Diamond‐Blackfan anaemia, ferritin, iron overload, liver iron content

## Abstract

In Diamond‐Blackfan anaemia (DBA), iron overload (IO) is common in transfusion‐dependent patients, yet has also been reported in non‐transfusion‐dependent patients. We explored the incidence of IO in transfusion‐dependent and non‐transfusion‐dependent DBA patients. We observed hepatic IO in 65% of patients analysed with MRI, including three patients that were only treated with transfusions in the past. Whereas overall ferritin levels and liver iron content correlated, ferritin levels did not reflect total body iron adequately. Our data suggest that transfusion burden in the past plays an important role in IO in DBA, and should be taken into account during follow up.

1

*Diamond‐Blackfan anaemia (DBA)* is an inherited bone marrow failure syndrome characterized by hypoplastic anaemia, congenital malformations and a predisposition to cancer [1]. Treatment of DBA consists of red blood cell (RBC) transfusions, glucocorticoids and, in a selection of patients, allogeneic haematopoietic stem cell transplantation. Whereas the majority of patients are glucocorticoid‐responsive at some point during the course of disease, approximately thirty percent of patients is dependent on regular RBC transfusions. Chronic RBC transfusions lead to iron overload (IO), which affects many organs, particularly the liver, heart and endocrine tissues [2]. Secondly, in certain hereditary anaemias, such as beta‐thalassemia and congenital dyserythropoietic anaemia, erythroferrone inappropriately stimulates enteral absorption of iron via hepcidin suppression, and thereby leads to IO [3, 4, 5]. To evaluate the iron status of patients, classically serum ferritin levels are determined, often with a cut‐off of >1000 ng/ml to initiate chelation therapy [6]. However, in different types of hereditary anaemia serum ferritin levels do not represent organ iron content accurately, and IO can be underestimated [7, 8]. Nowadays, magnetic resonance imaging (MRI) is therefore the golden standard for the diagnosis of IO in liver and heart [3, 9, 10].

In DBA, RBC transfusions are the main cause of IO [11]. Studies focusing on IO in non‐transfusion‐dependent are scarce, yet biochemical iron parameters suggesting IO in this population have been reported [12]. Previous studies have suggested that disturbed iron metabolism leads to an increased susceptibility to IO in DBA patients [12, 13, 14]. The impaired proliferation of erythroid progenitors limits the utilization of iron, resulting in an imbalance in the distribution of iron throughout the body and oversaturation of the organs that store iron.

Here we report the incidence and severity of IO in a well‐described cohort of transfusion‐dependent and non‐transfusion‐dependent DBA patients with the aim to improve the diagnostic evaluation and overall management of IO in DBA.

In this retrospective, observational multicentre study, we have included paediatric and adult DBA patients of whom serum ferritin level and/or MRI results were available. Patients were classified as transfusion‐dependent if they had received 10 or more RBC transfusions during the last 12 months prior to iron evaluation.‡ Non‐transfusion‐dependent patients were treated with glucocorticoids or received no treatment. Previous treatment and transfusion burden was assessed from medical records. Serum ferritin levels ≥250 ng/ml in males and ≥150 ng/ml in females were considered to be elevated. Results of MRI analyses were expressed as liver iron content (LIC) in mg/g and as cardiac T2* in milliseconds (ms). LIC ≥3 mg/g indicates significant hepatic IO, and LIC ≥7 mg/g is considered as moderate to severe IO [8]. Cardiac T2* ≤20 ms indicates significant cardiac IO [8, 9]. For patients analysed with MRI, iron parameters related to the date of the MRI were analysed. When no MRI analysis was available, the most recent iron parameters were analysed.

Twenty‐nine patients were included, including seventeen patients (17/29, 59%) in which MRI analysis of IO had been performed. The median age was 12 years (range 1–47 years), nine patients (9/29, 31%) were male and in twenty‐four (24/29, 83%) of the patients a molecular defect had been confirmed. Based on our criteria, ten patients (10/29, 34%) were transfusion‐dependent and nineteen (19/29, 66%) were non‐transfusion‐dependent. MRI results were available in nine of the transfusion‐dependent patients (9/10, 90%) and in eight of the non‐transfusion‐dependent patients (8/19, 42%). In the other patients, MRI analysis was not performed based on either clinical, biochemical or psychological reasons, assessed by the treating physician. Patient characteristics are summarized in Table 1.

**TABLE 1:** Patient characteristics including type of treatment and iron parameters

Patient	Gender	Age (years)	Affected gene	Current treatment	Chelation treatment	Previous treatment	Transfusion burden	Hb (g/dl)	MCV (fL)	Retic (*10ˆ9/L)	Ferritin (ng/ml)	TSAT (%)	LIC (mg/g)	Cardiac T2* (ms)
1	M	7	Unknown	T, GC	Yes	GC	≥10	6.5	94	39.7	534	41	19.7	27.5
2	M	6	Unknown	T, GC	Yes	GC	≥10	7.6	91	26.3	967	40	10.0	30
3	F	19	*RPS26*	T	Yes	GC, leucine	≥10	12.7	90	17.3	187	85	1.3	38.4
4	F	5	*RPS26*	T	Yes	GC	≥10	9.4	87	8.3	380	74	11.4	48.4
5	F	12	*RPS26*	T	Yes	GC, leucine	≥10	9.7	89	9.6	810	97	11.7	22
6	F	47	*RPL11*	T	Yes	GC	≥10	6.8	110	37.9	587	89	12.7	36
7	F	6	Unknown	T	Yes	GC	≥10	12.1	89	13.7	602	107	13.9	32
8	F	1	Unknown	T	None	None	≥10	7.6	87	24.4	599	30	NA	NA
9	F	13	*RPL5*	T	Yes	GC	≥10	10.8	92	17.6	856	NA	15.1	NA
10	F	4	*RPL5*	T	Yes	GC	≥10	9.4	90	15.4	637	89	12.5	49.5
11	F	9	*RPS7*	None	None	T, GC	1–10	12.4	90	70.5	50	18	0.8	40
12	M	22	*RPS17*	None	None	None	0	15.5	102	58.8	53	33	NA	NA
13	F	8	*RPS26*	None	None	GC	1‐10	12.7	93	48.9	28	33	NA	NA
14	F	26	*RPS19*	None	None	T, GC	≥10	11.1	101	81.5	277	51	7.3	33.7
15	F	10	*RPL35A*	None	None	T, GC	≥10	11.3	100	86.7	330	47	NA	NA
16	F	2	*RPL5*	None	None	None	0	10.8	85	91.4	36	24	NA	NA
17	M	8	*RPL9*	None	None	T, GC	≥10	11.3	88	42.9	181	33	3.9	20
18	F	13	*RPS19*	None	None	T, GC	1‐10	11.6	96	47.4	44	38	1.0	41
19	M	16	*RPS19*	None	None	T, GC	1‐10	14.5	90	38.0	46	23	0.9	NA
20	F	37	*RPL5*	None	None	Darbopoeietin, filgastrim	≥10	11.0	96	86	82	28	NA	NA
21	F	18	*RPL5*	GC*	None	T	≥10	6.9	100	70.8	263	56	4.0	35
22	F	16	*RPS26*	GC	None	T	1‐10	12.4	111	99.5	48	39	NA	NA
23	F	12	*RPS24*	GC	None	T	1‐10	11.6	100	41.5	55	36	NA	NA
24	M	12	*GATA1*	GC	None	T	≥10	9.4	107	94.8	65	37	NA	NA
25	F	18	Unknown	GC	None	T	≥10	12.1	103	43.8	173	15	NA	NA
26	F	18	*RPS16*	GC	None	T	1‐10	12.6	101	56.2	44	42	1.2	29
27	M	6	*RPS17*	GC	None	T	1‐10	13.9	90	51.2	46	21	NA	NA
28	M	14	*RPL11*	GC	None	T	1‐10	11.1	98	33	48	55	0.9	44
29	M	1	*RPS26*	GC	None	T	1‐10	11.0	104	108.5	190	NA	NA	NA

*Note*: All patients currently being treated with transfusions were considered as transfusion‐dependent (10/29 patients). All other patients were considered to be non‐transfusion‐dependent (19/29 patients).

Abbreviations: GC, glucocorticoids; Hb, haemoglobin; LIC, liver iron content; MCV, mean corpuscular volume; NA, not available; Retic, reticulocytes; T, transfusions; TSAT, transferrin saturation.

*Patient 21 did receive two RBC transfusions in the last 12 months prior to iron evaluation.

Hepatic IO was present in eleven (11/17, 65%) patients that were analysed by MRI. In the majority, (9/11, 82%), a LIC >7 mg/g indicated moderate to severe IO. (Figure 1A)

**FIGURE 1:**
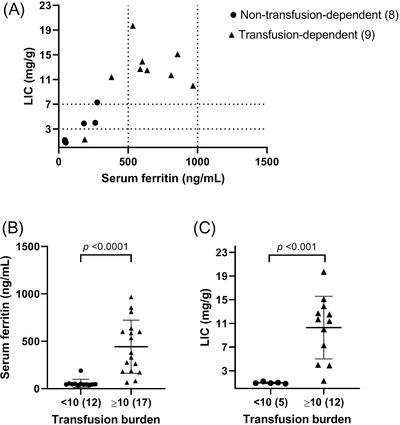
Parameters of iron overload in patients with Diamond‐Blackfan anaemia. (A) Serum ferritin levels and corresponding liver iron content (LIC) values per patient. (**B)** Serum ferritin levels grouped for transfusion burden, <10 transfusions and ≥10 transfusions. (**C)** LIC grouped for transfusion burden, <10 transfusions and ≥10 transfusions. LIC liver iron content

In the transfusion‐dependent‐group, eight patients (8/9, 89%) were diagnosed with moderate to severe hepatic IO, all were treated with chelation therapy. Interestingly, in the non‐transfusion‐dependent group, hepatic IO was present in three (patients 14, 17 and 21) patients (3/8, 38%) and none of these patients received chelation therapy. In this group, in one patient (patient 17), cardiac T2* was 20 ms, suggesting borderline cardiac IO. However, it should be noted that the image quality in this patient was suboptimal due to motion artefacts. As expected, comparing iron parameters between transfusion‐dependent and non‐transfusion‐dependent patients revealed a significant difference in both mean serum ferritin level (616 ng/ml vs. 108 ng/ml, *p* < 0.0001) and mean LIC value (12.03 mg/g vs. 2.50 mg/g, *p* < 0.001). (Supplementary Data Figure S1)

Based on transfusion history, patients were classified in three distinct groups: never transfused (2/29, 7%), 1–10 transfusions (10/29, 34%), and ≥10 transfusions (17/29, 59%). Of the patients evaluated with MRI (*n* = 17), all patients had been treated with RBC transfusions during the course of disease: five patients (5/17, 29%) had received less than 10 RBC transfusions, and twelve patients had received more than 10 RBC transfusions during their lives (12/17, 71%). Within this last group, eleven (11/12, 92%) patients were diagnosed with hepatic IO, in contrast to none in the group of patients who had received less than 10 transfusions. The non‐transfusion‐dependent patients with hepatic IO (patients 14, 17 and 21) were the only patients that received more than 10 RBC transfusions in the group of non‐transfusion‐dependent patients that underwent MRI analysis (12, 24, and 32 RBC transfusions respectively). Mean serum ferritin levels and mean LIC values were significantly higher in patients with a high transfusion burden (443 ng/ml vs. 57 ng/ml (*p* < 0.0001) and 10.29 mg/g versus 0.96 mg/g (*p* < 0.001), respectively) (Figure 1B,C).

As expected, we observed that overall serum ferritin levels and LIC values correlated significantly (*r* = 0.825, *p* < 0.001). Interestingly, while all transfusion‐dependent patients were treated with iron chelation therapy, and serum ferritin levels did not exceed 1000 ng/ml (Figure 1A; range 380–967 ng/ml), the majority of patients still suffered from moderate to severe hepatic IO (LIC ≥7 mg/g), indicating suboptimal management of IO. While serum ferritin levels are generally used to titrate chelation therapy, our data suggest that in DBA, similar to other types of hereditary anaemia, serum ferritin levels do not adequately reflect total body iron [7, 8]. Therefore, despite the significant correlation between serum ferritin levels and LIC values, this parameter cannot be used exclusively to screen for or clinically manage IO in DBA, as this may lead to an underestimation of the effect of chelation therapy on hepatic IO. In line with this, the three non‐transfusion‐dependent patients with hepatic IO had normal or only mildly elevated serum ferritin levels which would not prompt chelation therapy in clinical practice (277 ng/ml, 181 ng/ml and 263 ng/ml, respectively). Further analysis based on transfusion burden, illustrates that the number of transfusions discriminates between the patients with and without significant IO.

Since the need for treatment with RBC transfusions in DBA can vary during the course of disease, and a significant proportion of DBA patients is only treated with regular RBC transfusions during the first year of life, the total iron burden can be underestimated during clinical follow‐up and while interpreting iron status later in life. In particular in non‐transfusion‐dependent patients with long‐term stable disease, follow‐up may consist of infrequent visitations, and sequelae of IO and other DBA‐associated health issues can be overlooked. Whereas also in our population IO was evaluated with MRI in only 59% of patients, our data illustrate that non‐transfusion‐dependent DBA patients can be at risk to develop clinically significant IO.

In addition to transfusion burden in the past, IO in non‐transfusion‐dependent patients with hereditary anaemias, such as beta‐thalassemia and congenital dyserythropoietic anaemia, is often the result of ineffective erythropoiesis, where high levels of erythroferrone inhibit hepcidin production [5]. Although erythroferrone levels have not been investigated in DBA, it seems unlikely that it plays an important role due to the absence of increased erythropoiesis. Therefore it is likely that DBA patients are more susceptible to organ toxicity of IO due to relatively high levels of non‐transferrin bound iron and labile iron, as a result of limited iron uptake by erythroblasts [11, 13, 15].

In summary, the evaluation of IO in DBA requires critical analysis of biochemical parameters in combination with transfusion history, specifically in non‐transfusion‐dependent patients. Based on our data, we recommend to perform MRI‐based evaluation of IO in all DBA patients that were treated with regular RBC transfusions, and start chelation therapy early in treatment accordingly.

## FUNDING INFORMATION

The authors received no specific funding for this work.

## CONFLICT OF INTEREST

The authors declare no conflict of interest.

## AUTHOR CONTRIBUTIONS

JW and BD analysed data and wrote the manuscript. EH, FS, AV, AvdV and EB provided data and reviewed the manuscript. RW, WS and EN reviewed the manuscript. MB designed the study, analysed data and wrote the manuscript.

## ETHICS STATEMENT

This study was performed according to the Decleration of Helskinki and was approved by the medical ethical committee of all institutions. Data were obtained from the Diamond Blackfan Anaemia The Netherlands (DBAN) patient registry. Written informed consent was obtained from all patients and/or legal guardians.

## Supporting information

Figure S1. Serum ferritin and liver iron content in patients with Diamond‐Blackfan anaemia.

## Footnotes

‡ Various definitions of transfusion‐dependency are used in literature. In this study, the cut‐off of 10 RBC transfusions per twelve months was used, based on the study by Pospisilova et al.^12^

## References

[1] Bartels M , Bierings M . How I manage children with Diamond‐Blackfan anaemia. Br J Haematol. 2019;184(2):123–33. 30515771 10.1111/bjh.15701 PMC6587714

[2] Siah CW , Ombiga J , Adams LA , Trinder D , Olynyk JK . Normal iron metabolism and the pathophysiology of iron overload disorders. Clin Biochem Rev. 2006;27(1):5‐16. 16886043 PMC1390789

[3] Fleming RE , Ponka P . Iron overload in human disease. N Engl J Med. 2012;366(4):348–59. 22276824 10.1056/NEJMra1004967

[4] Porter JB . Practical management of iron overload. Br J Haematol. 2001;115(2):239–52. 11703317 10.1046/j.1365-2141.2001.03195.x

[5] Grootendorst S , de Wilde J , van Dooijeweert B , van Vuren A , van Solinge W , Schutgens R , et al. The interplay between drivers of erythropoiesis and iron homeostasis in rare hereditary anemias: tipping the balance. Int J Mol Sci. 2021;22 :2204. 33672223 10.3390/ijms22042204 PMC7927117

[6] Angelucci E , Barosi G , Camaschella C , Cappellini MD , Cazzola M , Galanello R , et al . Italian Society of Hematology practice guidelines for the management of iron overload in thalassemia major and related disorders. Haematologica. 2008;93(5):741–52. 18413891 10.3324/haematol.12413

[7] Taher A , El Rassi F , Isma'eel H , Koussa S , Inati A , Cappellini MD . Correlation of liver iron concentration determined by R2 magnetic resonance imaging with serum ferritin in patients with thalassemia intermedia. Haematologica. 2008;93(10):1584–6. 18728025 10.3324/haematol.13098

[8] van Straaten S , Biemond BJ , Kerkhoffs JL , Gitz‐Francois J , van Wijk R , van Beers EJ . Iron overload in patients with rare hereditary hemolytic anemia: evidence‐based suggestion on whom and how to screen. Am J Hematol. 2018;93(11):E374–6. 30105801 10.1002/ajh.25251 PMC6220762

[9] Wood JC . Guidelines for quantifying iron overload. Hematology Am Soc Hematol Educ Program. 2014;2014(1):210–5. 25696857 10.1182/asheducation-2014.1.210

[10] Viprakasit V , Ajlan A , Aydinok Y , Al Ebadi BAA , Dewedar H , Ibrahim AS , et al. MRI for the diagnosis of cardiac and liver iron overload in patients with transfusion‐dependent thalassemia: an algorithm to guide clinical use when availability is limited. Am J Hematol. 2018;93(6):E135–7. 29473204 10.1002/ajh.25075

[11] Roggero S , Quarello P , Vinciguerra T , Longo F , Piga A , Ramenghi U . Severe iron overload in Blackfan‐Diamond anemia: a case‐control study. Am J Hematol. 2009;84(11):729–32. 19810012 10.1002/ajh.21541

[12] Pospisilova D , Holub D , Zidova Z , Sulovska L , Houda J , Mihal V , et al. Hepcidin levels in Diamond‐Blackfan anemia reflect erythropoietic activity and transfusion dependency. Haematologica. 2014;99(7):e118–21. 24727814 10.3324/haematol.2014.104034 PMC4077099

[13] Berdoukas V , Nord A , Carson S , Puliyel M , Hofstra T , Wood J , et al. Tissue iron evaluation in chronically transfused children shows significant levels of iron loading at a very young age. Am J Hematol. 2013;88(11):E283–5. 23861216 10.1002/ajh.23543

[14] Porter JB , Walter PB , Neumayr LD , Evans P , Bansal S , Garbowski M , et al. Mechanisms of plasma non‐transferrin bound iron generation: insights from comparing transfused diamond blackfan anaemia with sickle cell and thalassaemia patients. Br J Haematol. 2014;167(5):692–6. 25209728 10.1111/bjh.13081 PMC4577015

[15] Li H , Lodish HF , Sieff CA . Critical issues in Diamond‐Blackfan anemia and prospects for novel treatment. Hematol Oncol Clin North Am. 2018;32(4):701–12. 30047421 10.1016/j.hoc.2018.04.005 PMC8162701

