# Supplementary material for: Transfusion burden in early childhood plays an important role in iron overload in Diamond‐Blackfan anaemia
Source: EJHaem. 2022 Aug 30;3(4):1300–4. doi: 10.1002/jha2.524 (PMC9713217; doi:10.1002/jha2.524)
Supplement: Supplementary file 1 — Figure S1. Serum ferritin and liver iron content in patients with Diamond‐Blackfan anaemia. [file JHA2-3-1300-s001.docx]

**Supplementary data.**


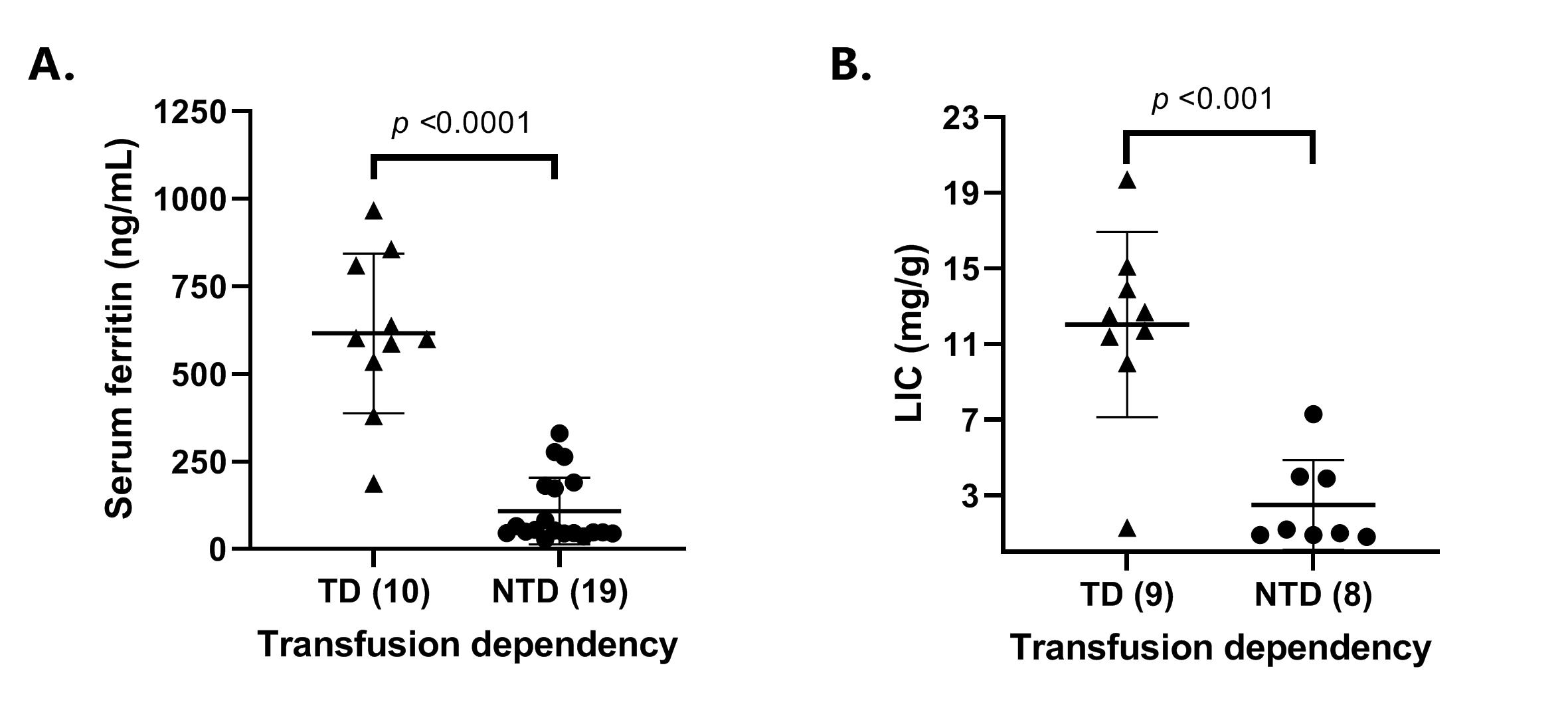


**Figure S1. Serum ferritin and liver iron content in patients with Diamond-Blackfan anaemia. A.** Serum ferritin levels grouped for transfusion-dependency (transfusion-dependent versus non-transfusion-dependent). **B.** Liver iron content grouped for transfusion-dependency (transfusion-dependent versus non-transfusion-dependent).
LIC liver iron content ; NTD non-transfusion dependent ; TD transfusion-dependent.
